# Synthesis and Characterization of Zirconia–Silica PMMA Nanocomposite for Endodontic Implants

**DOI:** 10.3390/dj11030057

**Published:** 2023-02-22

**Authors:** Puji Widodo, Wawan Mulyawan, Nina Djustiana, I. Made Joni

**Affiliations:** 1 Dentistry Department, Post Graduate School, Faculty of Dentistry, Universitas Padjadjaran, Jalan Sekeloa Selatan 1, Bandung 40132, Indonesia; 2 Functional Nano Powder University Center of Excellence, Universitas Padjadjaran, Jalan Raya Bandung-Sumedang KM 21, Jatinangor, Sumedang 45363, Indonesia; 3 Department of Community Medicine, Faculty of Medicine, Universitas Indonesia, Jalan Pegangsaan Timur 16, Cikini, Jakarta Pusat 10310, Indonesia; 4 Department of Dental Materials Science and Technology, Faculty of Dentistry, Universitas Padjadjaran, Jalan Raya Bandung-Sumedang KM 21, Jatinangor, Sumedang 45363, Indonesia; 5 Department of Physics, Faculty of Mathematics and Natural Sciences, Universitas Padjadjaran, Jalan Raya Bandung-Sumedang KM 21, Jatinangor, Sumedang 45363, Indonesia

**Keywords:** zirconia, silica, endodontic implant, PMMA

## Abstract

This study aimed to enhance the mechanical properties of PMMA composites by introducing various types of fillers, including ZrO_2_, SiO_2_, and a mixture of ZrO_2_-SiO_2_ nanoparticles, which were prepared as prototypes for an endodontic implant. The ZrO_2_, SiO_2_, and mixed ZrO_2_-SiO_2_ nanoparticles were synthesized using the sol–gel method and the precursors Tetraethyl Orthosilicate, Zirconium Oxychloride, and a mixture of both precursors, respectively. Before polymerization, the as-synthesized powders were subjected to the bead milling process to obtain a well-dispersed suspension. Two scenarios for the fillers were implemented in the preparation of the PMMA composite: a mixture of ZrO_2_/SiO_2_ and ZrO_2_-SiO_2_ mixed with two different types of silane: (3-Mercaptopropyl) trimethoxysilane (MPTS) and 3-(Trimethoxysilyl) Propyl Methacrylate (TMSPMA). The observation of the characteristics of all of the investigated fillers included the use of a particle-size analyzer (PSA), a Zeta-potential analyzer, FTIR, XRF, XRD, and SEM. The mechanical properties of the MMA composites, as prepared under various scenarios, were observed in terms of their flexural strength, diametrical tensile strength (DTS), and modulus of elasticity (ME). These levels of performance were compared with a PMMA-only polymer. Each sample was measured five times for flexural strength, DTS, and ME. The results showed that the best PMMA composite was SiO_2_/ZrO_2_/TMSPMA, as revealed by measurements of the flexural strength, DTS, and ME corresponding to 152.7 ± 13.0 MPa, 51.2 ± 0.6 MPa, and 9272.8 ± 2481.4 MPa, which are close to the mechanical properties of dentin. The viability of these PMMA composites, as measured up to day 7, was 93.61%, indicating that they are nontoxic biomaterials. Therefore, it was concluded that the PMMA composite created with SiO_2_/ZrO_2_/TMSPMA can be considered to be an acceptable endodontic implant.

## 1. Introduction

Recently, the incredibly rapid research and development of dental-based composite materials, resulting from many types of engineering materials with various types of polymers and fillers, along with the proper selection of coupling agents, have improved filler–matrix compatibility. Polymethyl methacrylate (PMMA), commonly known as “bone cement”, is widely used in orthopedic and trauma surgery [1]. PMMA has been widely studied as a biomaterial for dental implants because of its excellent properties as an insulator of heat and electricity and its resistance to biodegradation [2]. In addition, several studies have shown that PMMA has biocompatible properties and characteristics supporting the attachment of mesenchymal stem cells and osteoblasts, and they recommend it as a post or endodontic implant material [3,4]. However, PMMA has characteristics that present as drawbacks to its use as a dental implant, including a lower level of mechanical strength and a higher modulus of elasticity, as compared to alloys and ceramics, which, consequently, has a detrimental effect on its receiving masticatory loads [5,6,7]. 

The standard for mechanical properties in the manufacture of endodontic implants is determined by adjusting to post-endodontic requirements since the mechanical functionality is almost the same. Endodontic posts are used to assist in the restoration of teeth when undergoing endodontic treatment where the existing tooth structure is unable to support the restoration of the crown of the tooth [8,9,10]. Currently, there are various types of endodontic posts with various characteristics on the market. One of the most critical characteristics of endodontic posts is their mechanical properties, including their flexural strength and modulus of elasticity [11,12]. The mechanical properties must be similar to the strength of dentin from the occlusal direction, and they can spread evenly on the tooth’s root. Normal dentin has a flexural strength of 212.9 ± 41.9 MPa [13]; when dentin teeth were treated with endodontic treatment, i.e., using NaOCl irrigation, their flexural strength decreased to 135.72 ± 7.83 [14]. Other requirements of posts include corrosion resistance, adhesion, and biocompatibility. In addition, endodontic posts should have a radiopaque appearance and be easy to insert and remove, especially if repeated endodontic treatments are required [15].

It has long been known that PMMA used as an endodontic post offers good biocompatibility [16] and several beneficial properties, such as being transparent, colorless, easy to handle and process, low cost, and high surface-area-to-volume ratio [17]. However, PMMA alone is deficient in flexural strength, and it does not meet the requirements of endodontic implant application. Therefore, micro/nanofillers, such as SiO_2_ [18] and ZrO_2_ [19], have been commonly used to reinforce the PMMA composite, since the flexural strength of these fillers is much higher than that of the PMMA matrix. The flexural strength properties of the PMMA composite are considered effective in receiving stresses, depending on the type of reinforcing materials and the bond strength between the matrix and the reinforced materials. The bond strength between the reinforced materials and the matrix also depends on the morphology of the reinforced filler, such as being spherical or fibrous [20]. The flexural strength of the PMMA composite increases significantly to 143.498 MPa when reinforced with 3% ZrO_2_, and to 239.632 MPa when reinforced with 5% glass fiber and 3% ZrO_2_ [20]. ZrO_2_ has a high level of mechanical strength and good biocompatibility when used as a filler in composite materials. In restorative dentistry, ZrO_2_ is reinforced to improve the mechanical properties and osseointegration of a composite material to avoid stress-protective implants with bone tissue [21,22,23,24,25]. Therefore, many efforts have been made to improve the osteogenic activity of ZrO_2_ by introducing metal coatings [21,22], with significantly improved corrosion resistance or by providing surface modifications via various coupling agents to help its osteointegration process with bone [23,24]. Therefore, four representative silanes with different end groups were grafted on a Y-TZP (yttria-stabilized tetragonal zirconia polycrystal) surface to improve the osteogenic activity of zirconia-based materials as implants [24]. The different end groups of the silanes are γ-metha-cryloxypropyltrimethoxysilane (γ-MPS, with a vinyl end group), methyltrimethoxysilane (MTMS, with a methyl end group), 3-mercaptopropyltrimethoxy-silane (MPTS, with a mercapto end group), and 3-aminopropyltriethoxysilane (APTES, with an amino end group). In in vitro cell experiments, the mercapto- and amino-terminal silanes were found to promote osteogenic differentiation and mineralization by increasing osteocalcin (OCN) and osterix (OSX) expression levels, while the vinyl- and methyl-terminal silanes exhibited inhibitory effects on collagen type 1 (COL-1), Runt-related transcription factor 2 (Runx2), and OCN expressions. In general, chemical agents with their functional groups are used to prevent the problem of the agglomeration of ceramics powders in different synthesis methods, i.e., during the drying and calcination processes [25,26,27].

In addition, the reinforced material tends to agglomerate during the polymerization process, consequently causing a reduction in the bond strength of the filler and matrix [20]. Therefore, the distribution and stability of the nanoparticle suspension in the organic solvent become essential. A coupling agent is usually used to resolve agglomeration problems during the molding of composites [26,27,28]. Others introduced an additional process by bead milling to have reduced size distribution and promote stable suspension with a coupling agent on the ceramic powder surface [28]. Further, a silane of (3-(Methacryloyloxy)propyl)trimethoxysilane (TMSPMA) is used as a coupling agent for the PMMA composite to develop methacrylate polymer-based biodegradable hybrids for regenerative medicine with acceptable cytotoxicity test [29]. 

Many possible external factors influence the healing of endodontic implants, including commercial activities, military operations, tourism, and high-altitude entertainment. According to a study by Hirai et al., (2018), intermittent hypobaric hypoxia improves wound healing, periapical inflammation, and bone loss [30]. Sasaki et al., (2019), also reported that hypoxia conditions induced HIF-1α and Arg1, which are responsible for spontaneous wound healing [31]. Therefore, it is crucial to investigate the performance of the composite as an endodontic implant under normal oxygen supply compared with intermittent hypobaric hypoxia conditions.

The present study aims to enhance the mechanical properties of PMMA composites by introducing various types of fillers (ZrO_2_, SiO_2_, and ZrO_2_-SiO_2_) synthesized using the sol–gel method. Before polymerization, the as-synthesized powders were subjected to the bead milling process to obtain a well-dispersed suspension. The novelty of the present study was the comparison performance of the PMMA composite by applying two scenarios of additional fillers, a mixture of ZrO_2_/SiO_2_, and ZrO_2_-SiO_2_ with two different types of silanes, MPTS and TMSPMA, aimed to obtain an acceptable composite for the endodontic post. This study provided a scientific contribution to the formulation and preparation methods of a PMMA composite to enable application as a prototype candidate for endodontic implants. Additional proper fillers, such as ZrO_2_ and SiO_2_, and coupling agents will produce enhance the mechanical properties and biocompatibility of the composite. Thus, the as-prepared composite was also subjected to a biocompatibility evaluation via toxicity test. Furthermore, this PMMA composite will be subjected to in vivo tests under normoxia (normal oxygen supply) and intermittent hypobaric hypoxia conditions.

## 2. Materials and Methods

### 2.1. Materials

The chemicals for the SiO_2_ synthesis were Tetraethyl Orthosilicate (CAS: 78-10-4), Acetic Acid Glacial (CAS: 64-19-7 |100056), and Ethanol 98% (CAS: 628-97-7) received from (EMSURE^®^ ACS, ISO, Reag. Ph Eur, Merck, Rahway, NJ, USA), and PEG 400 (CAS: 25322-68-3) purchased from PT Brataco Chemical, Indonesia. The chemicals for the ZrO_2_ synthesis were Zirconium Oxychloride Octahydrate 99% AR (Loba Chemie PVT. Ltd., Mumbai, India, CAS: 13520-92-8), Ammonia Solution 25% (Merck, CAS: 631-61-8), and Aquadest (CAS: 7732-18-5). The chemicals for the ZrO_2_-SiO_2_ mix were obtained from both precursors of SiO_2_ and ZrO_2_. The chemicals for the composite were Methyl Methacrylate (stabilized for synthesis, Merck) and Benzoyl Peroxide (CAS: 94-36-0) for synthesis (Merck), and two different types of silane of (3-Mercaptopropyl) trimethoxysilane (MPTS, Sigma-Aldrich, St. Louis, MO, USA, CAS: 40372-72) and 3-(trimethoxysilyl) propyl methacrylate 98% (TMSPMA, Sigma-Aldrich, CAS: 97 2530-85).

### 2.2. Synthesis of Fillers

#### 2.2.1. Synthesis SiO_2_

SiO_2_ particles were synthesized using the sol–gel method referred to by Dubey et al. [32]. Tetraethyl Orthosilicate (TEOS) precursor of 2.2 mL was mixed with acetic acid of 2.3 mL and stirred for 10 min. A solution of PEG 400 5 wt% in 12 mL of ethanol was added to the mixture and stirred for 30 min. The solution was then subjected to 24 h of aging and drying at 100 °C until the powder was completely dried. The obtained powder was then calcinated at 500 °C for 3 h and continued at 900 °C for 3 h. Calcined powders frequently produce agglomerated particles in a larger size distribution of particles. Therefore, the as-prepared powder was subjected to a bead milling process of 20 wt.% in ethanol media to obtain a nano-sized distribution of SiO_2_ particles [24]. The size distribution and zeta potential of the SiO_2_ suspension were analyzed before and after bead milling using PSA (SZ-100, Horiba, Japan). The SiO_2_ suspension was filtered and dried at 100 °C until the powder was completely dried. The SiO_2_ powder after bead milling was subjected to the characterization of crystallinity, elemental, morphology and size distribution, and IR spectroscopy correspondingly using X-ray Diffraction (XRD, Bruker D8 Advance with Cu Kα with = 1.54060 Å), X-Ray Fluorescence (XRF, Rigaku NeX CG, Tokyo, Japan), and Scanning Electron Microscopy (SEM, SU3500 Hitachi, Tokyo, Japan, operating at 15 kV accelerating voltage) with image analysis software employed for particle size distribution (ImageJ, NIH Image, Bethesda, MD, USA, version 1.46r: Java 1.60_20; iD5 ATR, Nicolet iS5, Thermo Scientific, Waltham, MA, USA)

#### 2.2.2. Synthesis ZrO_2_

ZrO_2_ was synthesized using a precipitation method, according to Rudzani et al. [33]. Zirconia Oxychloride Octahydrate (6.445 g) was dissolved in 100 mL of distilled water and stirred for 30 min. The solution of NH_4_OH 10% *v*/*v* in distilled water was dropped dropwise into the solution until the pH of the solution reached pH 10. The precipitated suspension evaporated at 100 °C to obtain a dried powder. As-prepared ZrO_2_ powder was obtained after calcinating the dried powder at 1100 °C for 2 h. Similarly, ZrO_2_ powder that has undergone calcination often produces agglomerated particles into a larger size distribution of particles. Therefore, the as-prepared powder was subjected to a bead milling process of 20 wt.% in ethanol to obtain a well-dispersed ZrO_2_ nanosuspension [33]. The size distribution and zeta potential of the ZrO_2_ suspension were analyzed before and after bead milling using PSA (SZ-100, Horiba, Japan). The ZrO_2_ suspension was filtered and dried at 100 °C until the powder was completely dried. After bead milling, the ZrO_2_ powder was subjected to the characterization of crystallinity, elemental, and morphology correspondingly using XRD, XRF, and SEM with similar equipment for SiO_2_ characterization.

#### 2.2.3. Synthesis of the ZrO_2_-SiO_2_ Mixture

The ZrO_2_-SiO_2_ mixture was prepared by the precipitation method and the modified method introduced by Elsandika et al. [34]. ZrOCl_2_.8H_2_O (19.33 g) was dissolved in 300 mL of Aquadest by vigorously stirring for 1 h and named solution A. Separately, Tetraethyl Orthosilicate (TEOS) (12.5 mL) was dissolved in 12.5 mL of ethanol 98% and prescribed as solution B. Solution B was then dropped dropwise into solution A and stirred until a homogeneous solution was obtained. The mixture of solutions A and B was placed in a hotplate magnetic stirrer at 100 °C for 10 min. The solution of NH_4_OH 10% *v*/*v* in distilled water was dropped dropwise into the mixture of solution A and B until the pH of the solution reached pH 11 and precipitation occurred. The precipitated solution was then washed until pH 7 and dried for 24 h in a drying oven. The obtained powder was then calcined in a furnace at 1100 °C for 2 h. Similarly, mixed ZrO_2_-SiO_2_ powder can produce agglomeration of particles into a larger size distribution of particles when calcined. Therefore, the as-prepared powder was subjected to a bead milling process of 20 wt.% in ethanol media to obtain the nano-sized distribution of the particles in the ZrO_2_-SiO_2_ mixture. The size distribution and zeta potential of ZrO_2_-SiO_2_ mixed suspension were analyzed before and after bead milling using PSA (SZ-100, Horiba, Kyoto, Japan). The ZrO_2_-SiO_2_ mixed suspension was filtered and dried at 100 °C until the powder was completely dried. The mixed ZrO_2_-SiO_2_ powder was characterized in terms of crystallinity, elemental, and morphology, correspondingly using XRD, XRF, and SEM with similar equipment for SiO_2_ characterization.

### 2.3. Preparation of Nanocomposite

Two scenarios of the MMA composite were prepared, each using SiO_2_/ZrO_2_ or ZrO_2_-SiO_2_ mixed filler with two diverse silane coupling agents, for instance, MPTS or TMSPMA. The sample code names were SiO_2_/ZrO_2_/MPTS, SiO_2_/ZrO_2_/TMSPMA, ZrO_2_-SiO_2_ mixed/MPTS, and ZrO_2_-SiO_2_ mixed/TMSPMA, as presented in Table 1, and compared with pristine MMA composite. The SiO_2_/ZrO_2_/MPTS composite was prepared by mixing MMA with MPTS under a magnetic stirrer and subsequently mixed with SiO_2_/ZrO_2_ filler using a wt.% ratio, as per Table 1. The mixture was kept under a magnetic stirrer for 1 h to obtain homogeneous suspension and well-dispersed SiO_2_/ZrO_2_ filler in the MMA matrix.

**Figure 1:**
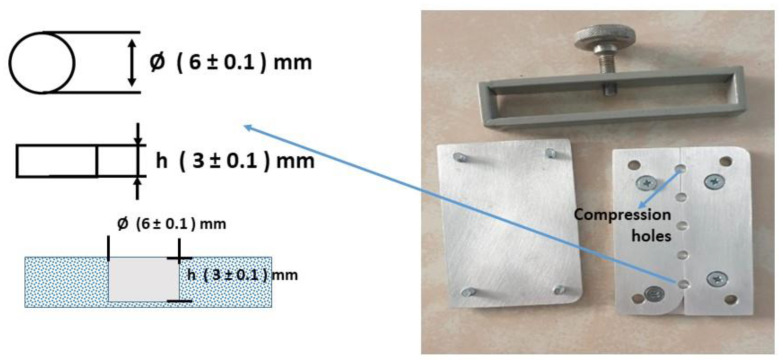
The compression molding of nanocomposite sample for DTS test.

**Figure 2:**
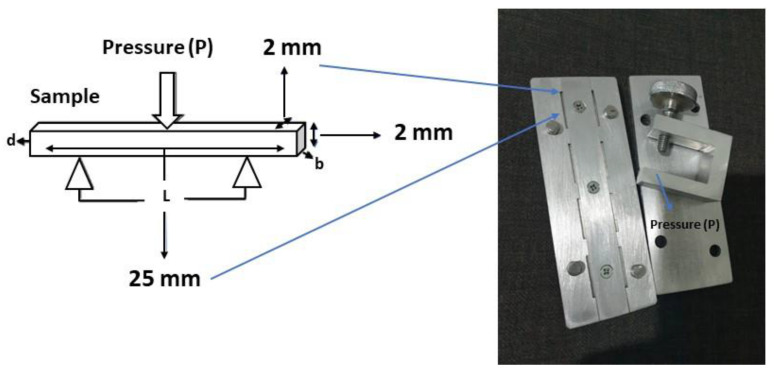
The compression molding of nanocomposite sample for flexural test and ME.

Before the preparation of the MMA composite, two types of molds were designed, each for DTS, as shown in Figure 1, and Flexural and ME, as shown in Figure 2. The mold was coated with Cold Mold Seal (CMS) before inserting the paste into the hole-shaped design. The polymerization was made by introducing an initiator of Benzoyl Peroxide into the suspension while keeping the suspension heated at 90 °C for 10 min until the solution turned into a paste. The paste was immediately poured into the peg mold and pressed to form the desired shape. The molding was placed in boiling water at 90 °C for 1 h, according to Wang et al. [35]. The other samples of SiO_2_/ZrO_2_/TMSPMA, ZrO_2_-SiO_2_ mixed/MPTS, and ZrO_2_-SiO_2_ mixed/TMSPMA were prepared with similar methods with different fillers and silanes. All composite samples were analyzed in terms of chemical bonding, crystallinity, and mechanical properties (DTS, Flexural, and ME) correspondingly using FTIR spectrometry (iD5 ATR, Nicolet iS5, Thermo Scientific, Waltham, MA, USA) in a wave number range of 4000–400 cm^−1^ at room temperature, X-ray Diffraction (XRD, Bruker D8 Advance with Cu Kα with = 1.54060 Å), and LLOYD Instrument (Type: LRX Plus, Ametek Company, Segenworth East Fareham, Hants, UK).

### 2.4. Toxicity Test

The dye exclusion test was used to determine the number of live cells present in the cell suspension. The toxicity test using the Doubling Time Trypan Blue protocol allows for light microscopic quantitation of cell viability. First, cells were suspended in PBS containing trypan blue and placed in the fibroblast cell line 3T3-L1 well plate (number of initial cell implantation = 250,000 cells/well). It is based on the principle that living cells have intact cell membranes that exclude certain dyes, such as Trypan Blue, Eosin, or Propidium, whereas dead cells do not. In this test, a cell suspension is mixed with a dye and visually examined to determine whether the cells are taking up or secreting the dye. In the protocol presented here, viable cells will have clear cytoplasm, whereas nonviable cells will have blue cytoplasm [36]. Cells suspended in PBS (Phosphate-Buffered Saline) containing Trypan Blue were then examined to determine the percentage of cells with clear cytoplasm (viable cells) versus cells with blue cytoplasm (nonviable cells) [36].

## 3. Results and Discussion

### 3.1. Characteristics of Filler before and after Beads Mill

**Figure 3:**
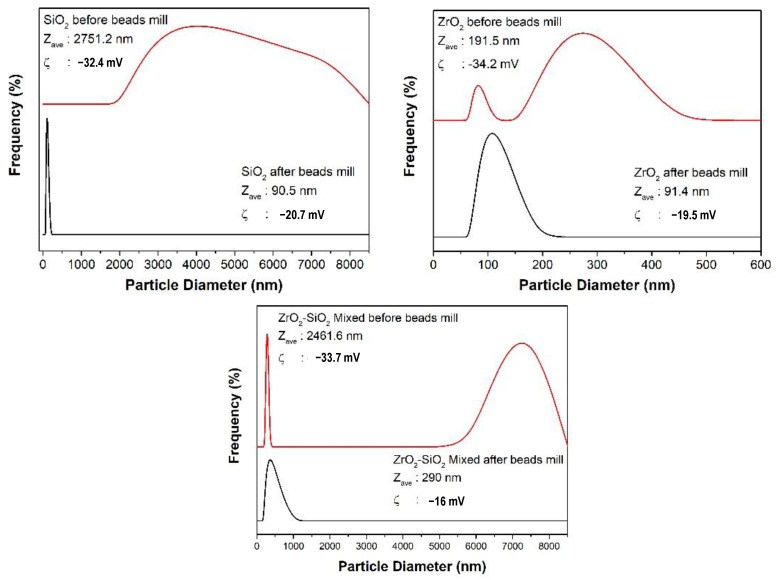
Particle size and stability of SiO_2_, ZrO_2,_ and ZrO_2_-SiO_2_ mixed filler solutions.

The size distribution of the synthesized particles before and after bead milling is presented in Figure 3. Particles appeared to agglomerate before bead milling, but agglomerated particles could be disintegrated after bead milling. In contrast, the nano-sized SiO_2_ and ZrO_2_ were obtained after bead milling, correspondingly, 90.5 and 91.4 nm. The ZrO_2_-SiO_2_ mixed particle was submicron sized (290 nm) after the bead milling process. The well-dispersed suspension was obtained for SiO_2_ nanoparticles due to the high zeta potential −20.7 mv to prevent deagglomeration of particles during the bead milling process. The suspension of ZrO_2_ and ZrO_2_-SiO_2_ mixed particles received lower zeta potentials, causing some particles to deagglomerate and form larger sizes during the bead milling process [37]. Consequently, the higher size distribution of both suspensions was obtained compared to the SiO_2_ suspension. 

**Figure 4:**
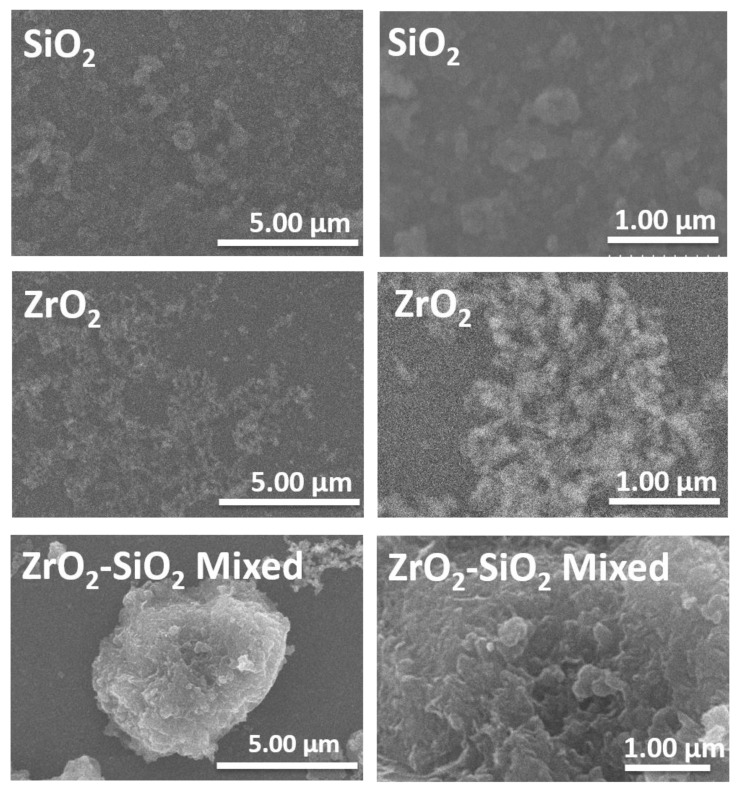
The SEM images of SiO_2_, ZrO_2,_ and ZrO_2_-SiO_2_ mixed fillers.

**Figure 5:**
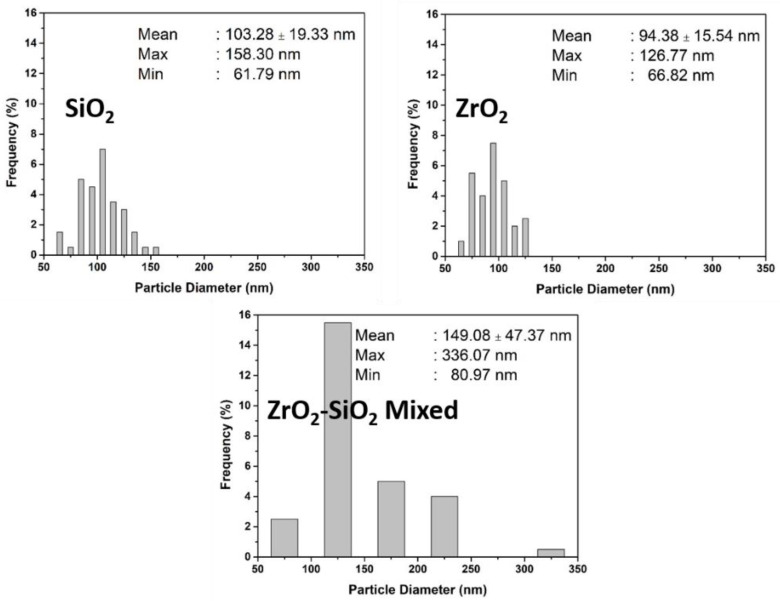
The Size Distribution of SiO_2_, ZrO_2,_ and ZrO_2_-SiO_2_ mixed fillers.

The SEM images were obtained to establish the morphology and also the corresponding size distribution of fillers after the bead milling, as shown in Figure 4 and Figure 5. The morphology of SiO_2_ and ZrO_2_ was spherical in shape, while the ZrO_2_-SiO_2_ mixed was relatively irregular. However, the corresponding size and size distribution of SiO_2_ and ZrO_2_ were higher than their suspension based on the PSA observation (Figure 3). Therefore, the spherical morphology of SiO_2_ and ZrO_2_ nanoparticles agrees with our previous study [38].

Based on the elemental analysis of XRF, the purity of SiO_2_ and ZrO_2_, and ZrO_2_-SiO_2_ mixed was, correspondingly, 98 and 83.7, as presented in Table 2. Meanwhile, the ZrO_2_-SiO_2_ mixed particles contained 31.7% mass of SiO_2_ and 62.7% mass of ZrO_2_. Several other impurities of minerals appeared, with the highest content being the Mg element. 

**Figure 6:**
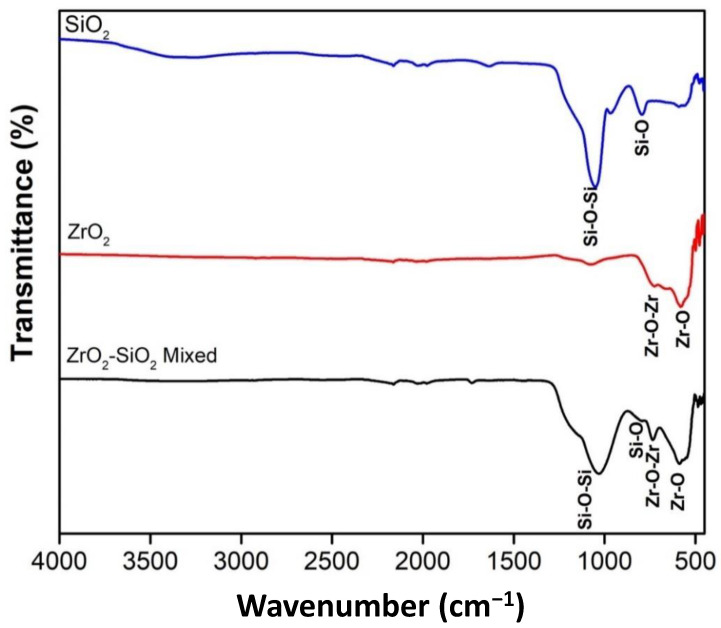
FTIR Filler SiO_2_, ZrO_2_, and ZrO_2_-SiO_2_ mixed.

Figure 6 shows the FTIR SiO_2_, ZrO_2_, and ZrO_2_-SiO_2_ mixed after the bead milling process. The FTIR of SiO_2_ fine powder has two prominent characteristic peaks, observed at about 793 cm^−1^ and 1050 cm^−1^, namely Si-O bending vibration band and asymmetric stretching vibration siloxane bonds (Si-O-Si) [38]. This result confirmed the formation of SiO_2_ and its functional groups via sol–gel synthesis. SiO_2_ nanopowder was produced after undergoing the sol–gel process with typical reactions of hydrolysis and condensation of precursors. The TEOS precursor was first hydrolyzed to silicic acid during the sol–gel process. Then, the condensation reaction led to the formation of Si-O-Si bonds. The high deformability of the formation allows the inorganic gel to accommodate significant shrinkage, which prevents cracking and pulverization of the powder during drying and calcination. 

The FTIR of ZrO_2_ shows a distinct peak attributed to Zr-O-Zr and Zr-O bonds. Similarly, the formation of zirconia was confirmed by the FTIR observation, indicating a successful precipitation process. In addition, several Si and Zr atoms appear in the FTIR of the ZrO_2_-SiO_2_ mixed sample, attributed to Si-O-Si, Si-O, and Zr-O bonds. This spectrum indicated the presence of the formation of zirconia and silica in the mixture. 

**Figure 7:**
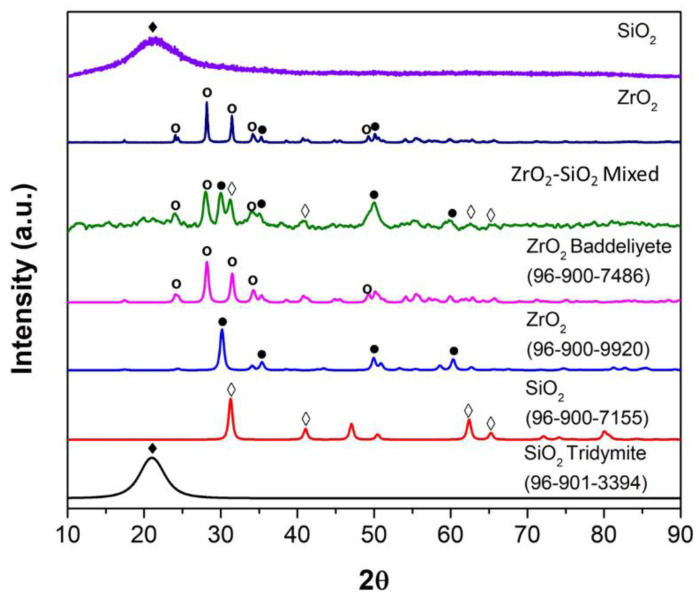
XRD Filler SiO_2_, ZrO_2_, and ZrO_2_-SiO_2_ mixed. XRD spectra references are SiO_2_ Tridymite/COD 96-900-7155 (solid square), SiO_2_ Stishovite/COD 96-900-7155 (hollow square), ZrO_2_ orthorhombic/COD 96-900-9920 (solid circle) and ZrO_2_ Baddeleyite/COD 96-900-7486 (hollow circle).

Figure 7 shows the XRD spectrum of the SiO_2_, ZrO_2_, and ZrO_2_-SiO_2_ mixed samples compared with the reference spectrum based on Crystallography Open Database from Match version 3.6.2.121 software Crystal Impact, Bonn, Germany. The crystal structure of the SiO_2_ sample appeared to be triclinic (anorthic) Tridymite based on COD 96-901-3394, while O_2_ appeared to be a monoclinic Baddeleyite based on COD 96-900-7486. Meanwhile, the crystal structure of ZrO_2_-SiO_2_ mixed appeared to be a combination of crystalline tetragonal SiO_2_ Stishovite (COD 96-900-7155), monoclinic ZrO_2_ Baddeleyite (COD 96-900-7486), and orthorhombic ZrO_2_ (COD 96-900-9920), with ratios of 46%, 37.3%, and 16.7%, respectively.

### 3.2. Characteristics of Composite with Various Types of Fillers

**Figure 8:**
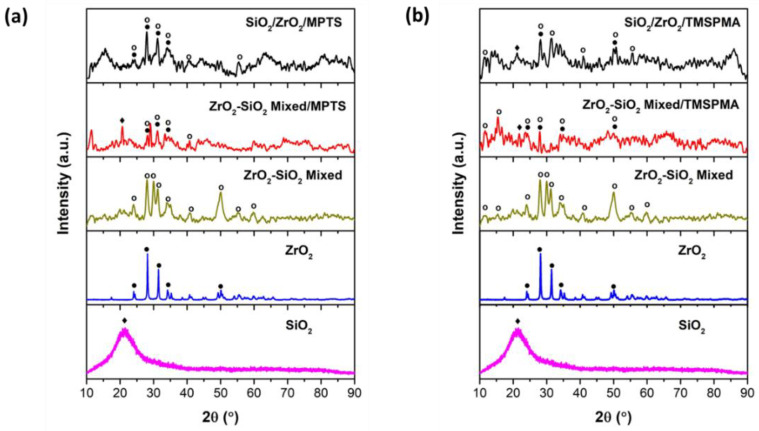
XRD composite post with silane addition: (**a**) MPTS and (**b**) TMSPMA. XRD spectra references are SiO_2_ Tridymite/COD 96-900-7155 (solid square) and ZrO_2_ Baddeleyite/COD 96-900-7486 (solid circle).

Two scenarios of the MMA composite were prepared and subjected to XRD observation where each composite used SiO_2_/ZrO_2_ or ZrO_2_-SiO_2_ mixed filler, with two diverse silane coupling agents, MPTS or TMSPMA. Figure 8 shows the XRD results of an endodontic implant composite with the addition of MPTS and TMSPMA silanes, which were analyzed using the Match version 3.6.2.121 software Crystal Impact, Bonn, Germany. In Figure 8a, the SiO_2_/ZrO_2_/MPTS sample has high peaks compared to ZrO_2_-SiO_2_ mixed/MPTS. The XRD pattern of both scenarios with high composite crystallinity indicates that the presence of fillers is well distributed in the polymer matrix. 

**Figure 9:**
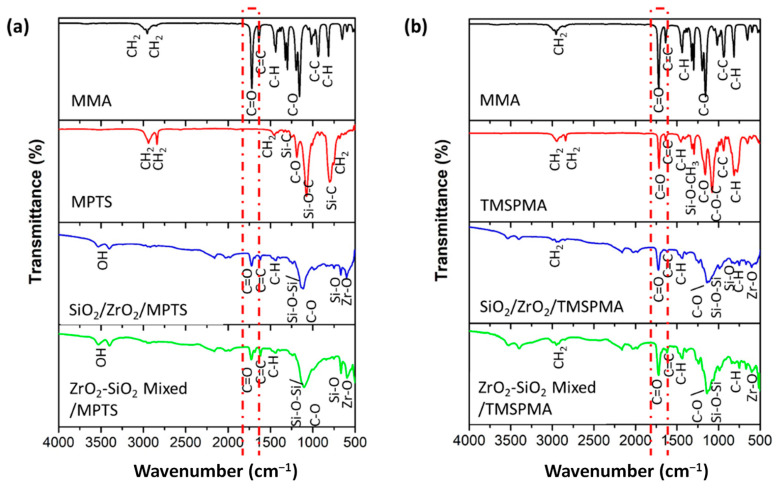
FTIR composite post with silane addition: (**a**) MPTS and (**b**) TMSPMA.

Figure 9a shows that the SiO_2_/ZrO_2_ composite with the MPTS surfactant did not show a strong bond, as shown by the weak peaks of the MPTS functional groups in the composite. The peaks represent that the TMSPMA surfactant had a stronger bond with the MMA matrix than MPTS for both composites with different fillers (SiO_2_/ZrO_2_ and ZrO_2_-SiO_2_ mixed). The strong C = O bond at 1723.03 cm^−1^ (dashed red box) for the composite with the TMSPMA surfactant is considered to be responsible for a strong filler–MMA matrix. The C = O asymmetric stretch is a characteristic of methacrylate formed in both composites. Other functional groups at 1205.44 cm^−1^ were assigned to Si-O-CH3 (Si-O bending). The wavenumber range for common functional groups in Figure 9 is summarized in Table 3.

### 3.3. Mechanical Properties of Composite with Various Types of Fillers

**Figure 10:**
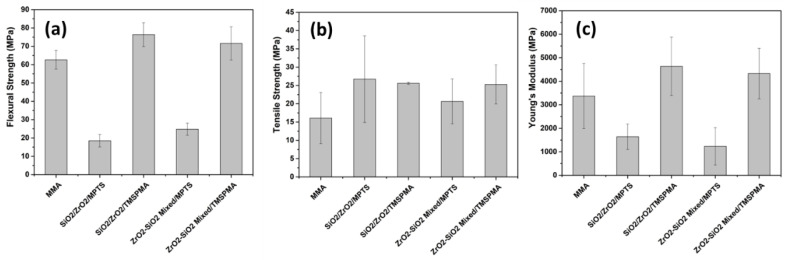
(**a**) Flexural, (**b**) DTS, and (**c**) Modulus of Elasticity.

Each sample was measured five times for flexural, DTS, and modulus of elasticity. Based on the results of mechanical tests on several samples, SiO_2_/ZrO_2_/ TMSPMA was better than the other samples seen from the flexural, DTS, and ME tests, with flexural values of 152.7 ± 13.0 MPa, DTS with values of 51.2 ± 0.6 MPa and ME of 9272.8 ± 2481.4 MPa (Figure 10). However, the mechanical properties of the composites obtained from MMA with MPTS were even lower than those of the MMA matrix without filler. This shows that MPTS is unsuitable as the MMA surfactant in the two fillers used. In contrast, the composite with TMSPMA showed enhanced mechanical properties compared to the composite with MPTS in both types of fillers (SiO_2_/ZrO_2_ and ZrO_2_-SiO_2_ mixed). It was highlighted that the composite with TMSPMA using SiO_2_/ZrO_2_ filler possessed higher mechanical properties than ZrO_2_-SiO_2_ mixed. The size and size distribution of SiO_2_/ZrO_2_ particles were lower compared to ZrO_2_-SiO_2_ mixed and so considered responsible for the strong bonding between the filler and matrix, causing stronger mechanical properties.

The SiO_2_/ZrO_2_/TMSPMA sample has a higher flexural value and a reasonably large DTS value than the other samples. In contrast, the SiO_2_/ZrO_2_/TMSPMA composite produced the highest modulus elastic properties. The mechanical properties of the composite are considered to be an endodontic implant when the mechanical properties are close to the dentin properties in terms of modulus elasticity and flexural strength. The modulus elasticity of general dentin is (17.5 ± 3.8) GPa [13], while the modulus elasticity of dentin in the anterior teeth is (5.3 ± 1.6) to (6.1 ± 1.6) MPa [39]. The flexural strength of dentin is 212.9 ± 41.9 MPa [13], while the flexural dentin in teeth after root canal treatment with saline irrigation after 30 days is 203.26 ± 16.76 [14]. The flexural dentin after root canal irrigation with NaOCl 5% is (135.72 ± 7.83)–(200 ± 8.66) MPa [14]. The as-prepared best composite with PMMA-SiO_2_/ZrO_2_/TMSPMA resulted in higher flexural strength than only PMMA with a Bis GMA base (83.5 ± 10.7 MPa), with an elastic modulus (5.0 ± 2.9) MPa [6]. Further, the flexural strength of the as-prepared SiO_2_/ZrO_2_/TMSPMA composite was higher compared to another study on a PMMA–silica nanofiber dental composite (132.74 ± 20.70) MPa [39]. In contrast, the as-prepared SiO_2_/ZrO_2_/TMSPMA composite showed excellent DTS compared to other reported PMMA composites using silica filler, with and without fiberglass, correspondingly (38.74 ± 3.05) and (28.39 ± 3.21) MPa [40]. It was emphasized that, based on the flexural strength, elastic modulus, and DTS, the PMMA composite with SiO_2_/ZrO_2_/TMSPMA is considered acceptable for an endodontic implant.

### 3.4. Toxicity Test of Selected Composite with Enhanced Mechanical Properties

**Figure 11:**
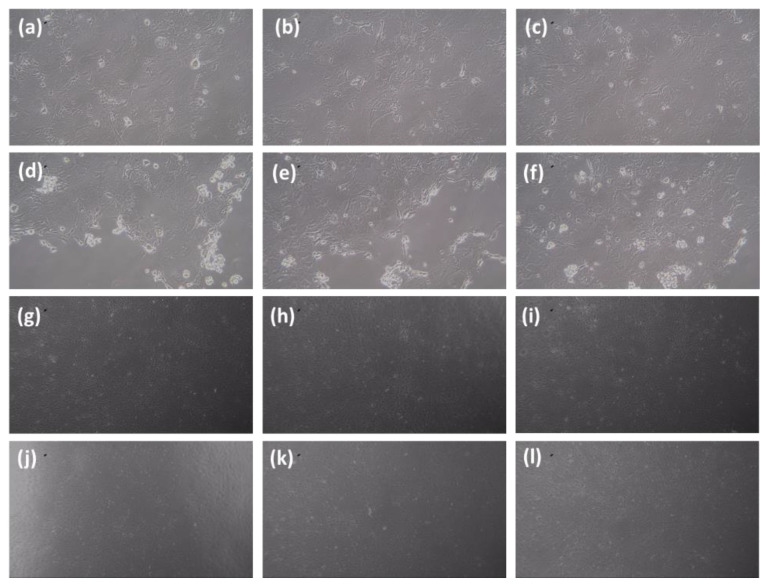
Microscopic image of 3T3-L1 Fibroblast cells with control (**a**–**c**) and SiO_2_/ZrO_2_/TMSPMA post composite (**d**–**f**) incubation period of 1 day, (**g**–**i**) incubation period of 4 days, and (**j**–**l**) 7-day incubation period.

The selected composite of SiO_2_/ZrO_2_/ TMSPMA with enhanced mechanical properties was subjected to a toxicity test as one of the essential composite criteria as an endodontic post or implant. Figure 11 shows a microscopic image of 3T3-L1 Fibroblast cells with control (a, b, c) and SiO_2_/ZrO_2_/TMSPMA post composite (d, e, f), incubation period of 1 day (g, h, i), incubation period of 4 days, and (j, k, l) 7 days incubation period. Based on the microscopic images, the viability of the living cell can be determined when the cell nucleus absorbs the blue color from trypan blue, according to the procedure carried out by Strober W, 2015 [36]. The cell is considered a living cell when it does not absorb the trypan blue color, the cell periphery glows, and the shape is perfectly round. On the other hand, the cell is considered to be dead when it is not perfectly round, and the cell wall is destroyed.

**Figure 12:**
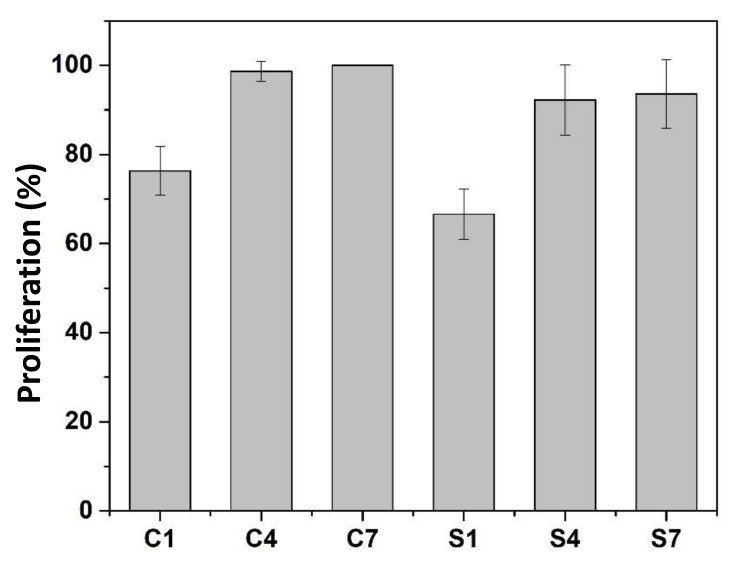
The positive cell growth curves at incubation periods of 1, 4, and 7 days, respectively, control 1 day (C1), control 4 days (C4), control 7 days (C7), sample SiO_2_/ZrO_2_/TMSPMA 1 day (S1), SiO_2_/ZrO_2_/TMSPMA 4 days (S4), and SiO_2_/ZrO_2_/TMSPMA 7 days (S7).

Figure 12 shows that the percentage of live cells (treated with control, C1, C4, and C7) on the first day of incubation (S1) was 87.06%, on the fourth day of incubation (S4) was 93.38%, and on the seventh day of incubation (S7) was 93.61%. The cytotoxicity level of each concentration of antibacterial agent was calculated as a percentage of cell viability, including absorbance values obtained for each system. According to ISO 10993-5, the percentage of cell viability was above 80%. Thus, it is considered non-cytotoxic based on the criteria that within 60–80% is weak, 40–60% moderate cytotoxicity, and below 40% severe cytotoxicity [41,42].

The endodontic implant obtained from the PMMA-ZrO_2_/SiO_2_-TMSPMA composite could improve its mechanical property close to dentin with good biocompatibility. Thus, this composite offers the possibility for clinical application since it can technically be produced in large quantities at an affordable cost. Further, theoretically, the presence of SiO_2_ and ZrO_2_ layers makes the composite non-corrosive and good at osteointegration with bone. Although further research is needed, particularly on the long-term biocompatibility properties, the recently existing properties meet the requirements for implants and endodontic posts.

## 4. Conclusions

The selected composite of SiO_2_/ZrO_2_/TMSPMA was considered acceptable as an endodontic implant in terms of the flexural strength, DTS, and elastic modulus, correspondingly (152.7 ± 13.0) MPa, (51.2 ± 0.6) MPa and (9272.8 ± 2481.4) MPa. The selected composite was also considered non-cytotoxic according to ISO 10993-5, with a percentage of cell viability above 80%. It was emphasized that the application of ZrO_2_/SiO_2_ nanofillers with a suitable and proper amount of silane coupling agent of TMSPMA enhanced the mechanical properties of PMMA to meet the criteria of the composite as an endodontic implant. However, the limitation of the present work is the biocompatibility, observed for only 14 days, so more extended periods are necessary. In addition, this result enabled an extended clinical test study to determine the healing rate of inflammation after implant placement, even observing the healing process during the induction of intermittent hypobaric hypoxia.

## Figures and Tables

**Table 1:** Experimental setup of the composite samples in comparison with only MMA.

No	Samples Code	Matrix MMA (wt.%)	Coupling Agent Silane (wt.%)		Initiator BP (wt.%)	Filler (wt.%)		
			MPTS	TMSPMA		ZrO_2_	SiO_2_	ZrO_2_-SiO_2_ Mixed
1	MMA	98.5	-	-	1.5	-	-	-
2	SiO_2_/ZrO_2_/MPTS	83	0.75	-	1.25	3.75	11.25	-
3	SiO_2_/ZrO_2_/ TMSPMA	83	-	0.75	1.25	3.75	11.25	-
4	ZrO_2_-SiO_2_ mixed/MPTS	83	0.75	-	1.25	-	-	15
5	ZrO_2_-SiO_2_ mixed/TMSPMA	83	-	0.75	1.25	-	-	15

MMA = Methyl Methacrylate; MPTS = 3-(Mercaptopropyl) trimethoxysilane; TMSPMA = 3-(trimethoxysilyl) propyl Methacrylate; BP = Benzoyl peroxide.

**Table 2:** XRF Filler SiO_2_, ZrO_2,_ and ZrO_2_-SiO_2_ mixed.

Component	SiO_2_	ZrO_2_	ZrO_2_-SiO_2_ Mixed
	(mass%)	(mass%)	(mass%)
Al	ND	3.390	1.670
Au	0.001	ND	ND
Bi	0.010	0.012	ND
Ca	0.028	0.012	0.038
Co	ND	0.026	ND
Cr	0.000	0.001	0.002
Cu	0.001	0.115	ND
Fe	0.005	0.033	0.005
Ga	ND	0.121	ND
K	0.028	ND	0.018
Mg	1.820	12.600	3.830
Pb	ND	0.012	ND
Pt	0.001	0.018	ND
S	0.002	ND	ND
Sn	0.001	0.012	0.012
Ti	0.004	ND	0.003
Zn	0.014	ND	0.025
SiO_2_	98	ND	31.7
ZrO_2_	0.090	83.7	62.7

**Table 3:** The wavenumber range for common functional groups.

Samples Code	Wavenumber (cm^−1^)	Assignment	Functional Group Name	Samples Code	Wavenumber (cm^−1^)	Assignment	Functional Group Name
MMA	2982.59	CH_2_	Alkane	SiO_2_/ZrO_2_/MPTS	3530.39	OH	hydroxyl
	2953.85	CH_2_	Alkane		1722.46	C = O	Carbonyl
	1719.98	C = O	Carbonyl		1684.25	C = C	Alkene
	1637.01	C = C	Alkene		1434.77	C-H	Alkane
	1437.75	C-H	Metyl		1114.06	Si-O-Si	Siloxane
	1157.50	C-O	Ester		1114.06	C-O	Ester
	938.66	C-C	Alkene		669.30	Si-O	Silanol
	814.86	C-H	Alkene		600.88	Zr-O	Zirconia
MPTS	2939.65	CH_2_	Alkane	ZrO_2_-SiO_2_ mixed/MPTS	3530.38	OH	Hydroxyl
	2838.98	CH_2_	Alkane		1725.70	C = O	Carbonyl
	1455.22	CH_2_	Alkane		1618.71	C = C	Alkene
	1257.78	SiC	Silicon carbide		1435.17	C-H	Alkane
	1188.16	C-O	Ester		1103.81	Si-O-Si	Siloxane
	1074.31	Si-O-C	Siloxane		1103.81	C-O	Ester
	800.71	Si-C	Silicon carbide		667.32	Si-O	Silanol
	753.42	CH_2_	Alkene		596.10	Zr-O	Zirconia
TMSPMA	2945.83	CH_2_	Alkane	SiO_2_/ZrO_2_/ TMSPMA	2949.29	CH_2_	Alkane
	2840.60	CH_2_	Alkane		1723.03	C = O	Carbonyl
	1716.56	C = O	Carbonyl		1619.85	C = C	Alkene
	1636.83	C = C	Alkene		1435.15	C-H	Alkane
	1295.44	Si-O-CH_3_	Methoxysilyl		1138.27	Si-O-Si	Siloxane
	1160.59	C-O	Ester		1113.74	C-O	Ester
	1077.23	C-O-C	Eter		750.19	Si-O	Silanol
	939.16	C-C	Alkene		669.00	C-H	Alkene
	813.12	C-H	Alkene		601.48	Zr-O	Zirconia
				ZrO_2_-SiO_2_ mixed/TMSPMA	2949.80	CH_2_	Alkane
					1721.10	C = O	Carbonyl
					1619.54	C = C	Alkene
					1435.26	C-H	Alkane
					1139.12	C-O	Ester
					1139.12	Si-O-Si	Siloxane
					668.51	C-H	Alkene
					600.75	Zr-O	Zirconia
